# Reactivation of Cytomegalovirus Increases Nitric Oxide and IL-10 Levels in Sepsis and is Associated with Changes in Renal Parameters and Worse Clinical Outcome

**DOI:** 10.1038/s41598-019-45390-x

**Published:** 2019-06-21

**Authors:** Taylon Felipe Silva, Virgínia Márcia Concato, Fernanda Tomiotto-Pellissier, Manoela Daiele Gonçalves, Bruna Taciane da Silva Bortoleti, Eliandro Reis Tavares, Lucy Megumi Yamauchi, Cintia Magalhães Carvalho Grion, Andréa Name Colado Simão, Milena Menegazzo Miranda-Sapla, Idessania Nazareth Costa, Wander Rogério Pavanelli, Ivete Conchon-Costa

**Affiliations:** 1 Laboratory of Immunoparasitology of neglected diseases and cancer - LIDNC, Department of Pathological Sciences, Center of Biological Sciences, State University of Londrina, Paraná, Brazil; 2 Laboratory of Biotransformation and Phytochemistry, Department of Chemistry, Center of Exact Sciences, State University of Londrina, Paraná, Brazil; 3 Department of Microbiology, Center of Biological Sciences. State University of Londrina, Paraná, Brazil; 4 Department of Clinical Medicine, Health Sciences Center, State University of Londrina, Paraná, Brazil; 5 Department of Pathology, Clinical Analysis and Toxicology, State University of Londrina, Paraná, Brazil; 6 Biosciences and Biotechnology Postgraduate Program, Carlos Chagas Institute (ICC), Fiocruz, Curitiba, Brazil

**Keywords:** Clinical microbiology, Infection, Viral infection

## Abstract

CMV reactivation has been widely associated with bacterial sepsis and occurs in approximately 30% of these individuals, is associated with a longer ICU stay, prolongation of the need for mechanical ventilation, and over 80% increase in the mortality rate, being directly associated with severe organ dysfunction and hemodynamic imbalance. Thus, the aim of this study was to evaluate the role of CMV reactivation in sepsis progression. The overall occurrence of cytomegalovirus reactivation in the cohort was 17.58%. Was observed an increase in plasma levels of NO, reduction of percentage of free days of mechanical ventilation and arterial pH, as well as changes in coagulation parameters in the reactivated group. There was also a significant increase in IL-10, creatinine, urea levels and reduction of 24-hour urine output. These variables still correlated with viral load, demonstrating an association between the reactivation process and kidney failure present in sepsis. The reactivated group still had 2.1 times the risk of developing septic shock and an increase in the mortality rates. CMV is reactivated in sepsis and these patients presented a higher risk of developing septic shock and higher mortality rates and our data suggest that IL-10 and NO may be involved in this process.

## Introduction

Sepsis is a pathological syndrome with biochemical abnormalities induced by infection. It is defined as an organic dysfunction with imminent threat to life triggered by a deregulated host response to an infection. It persists among the most serious worldwide public health issues with a growing incidence over the years. It is characterized by a heterogeneous host response to a pathogen amplified by several endogenous factors, leading to alterations in several physiological processes, such as cardiovascular, neurological, endocrine, metabolic, and coagulation. This broad perspective of changes define the biological significance and clinical heterogeneity of the affected individuals^1^.

Human Cytomegalovirus (CMV) is a double-stranded DNA virus that can be transmitted by various bodily secretions and fluids, such as saliva, sexual contact, blood transfusion, and transplantation of solid organs or bone marrow. Primary infection in immunocompetent individuals is generally asymptomatic and may result in a latent long-term infection condition is established, which may be reactivated in certain situations^2^.

CMV reactivation is an important aspect related to the pathogenesis of this viral infection. Immunosuppression, inflammation, infection and oxidative stress are important risk factors that may trigger reactivation. The exact mechanism required is yet to be fully elucidated, but studies have suggested the role of TNF-α as an important mediator to this process^3^.

The CMV reactivation has been widely associated with bacterial sepsis and is likely to result from the inflammatory process which is peculiar to such a condition^4^. Reactivation occurs in approximately 30% of individuals with sepsis without immunosuppression and is associated with a longer stay in ICU patients, greater need for mechanical ventilation^5^, and over 80% increase in the mortality rate^6^. In addition, CMV reactivation in patients with sepsis has been described as a worsening factor for clinical prognosis, with a significant increase in morbidity and mortality, directly associated with severe organ dysfunction and hemodynamic imbalance^7^. Thus, our aim was to evaluate the role of CMV reactivation and its association with immunological mediators, laboratory parameters, and clinical manifestations related to the prognosis and clinical outcome of patients with sepsis.

## Results

### Characteristics and prognostic scores of the patients

**Figure 1:**
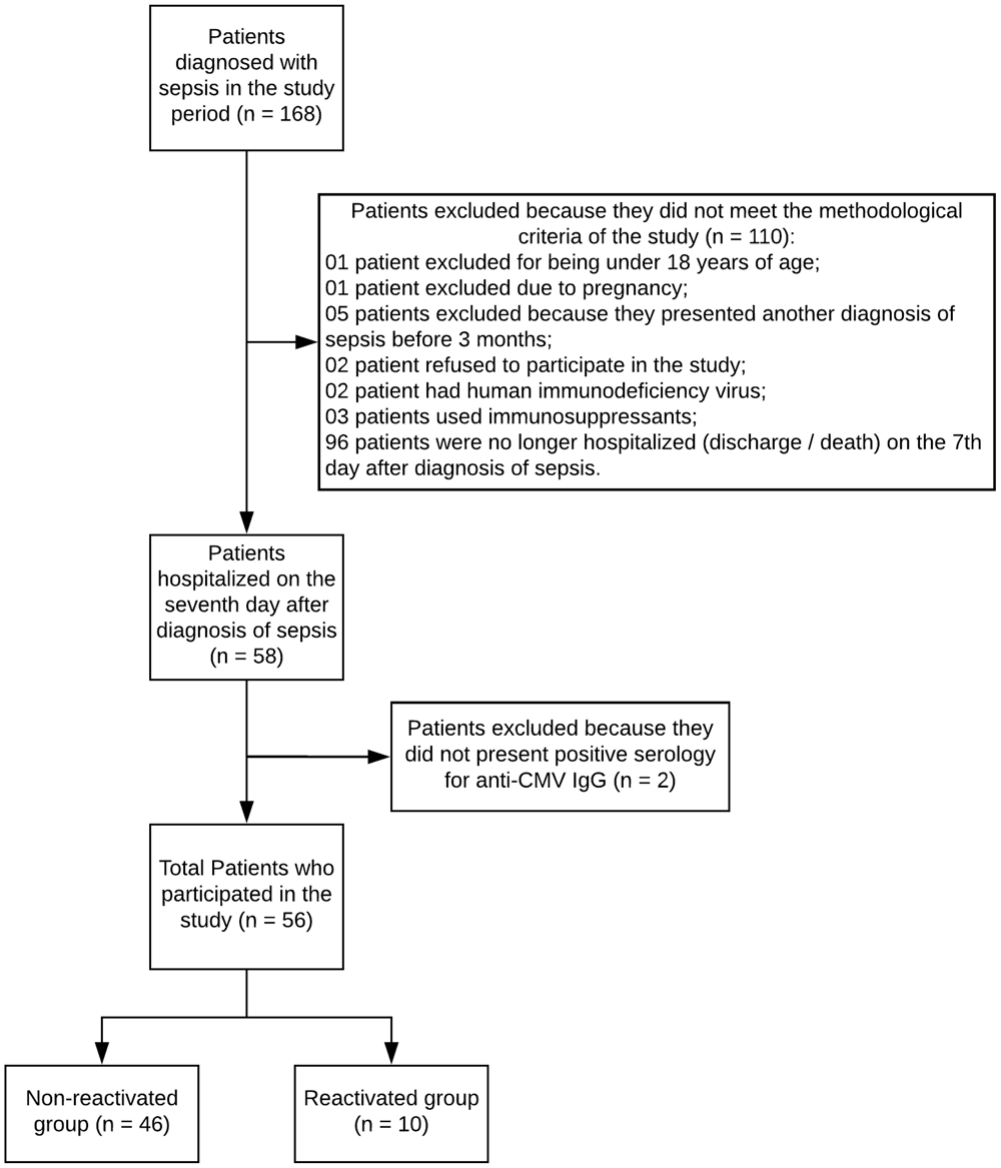
Methodological flow chart. Demonstration of patients excluded/included in the study and distribution of the groups evaluated.

During the study period, 168 patients were diagnosed with sepsis and evaluated regarding the inclusion and exclusion criteria. 110 patients were excluded for having not met the established criteria. Two out of the 58 patients who were still hospitalized on the seventh day after sepsis diagnosis did not present positive CMV serology and were therefore excluded. This generated a final sample of 56 patients, including 10 (reactivated group) who had detectable CMV viral load compatible with the reactivation process as described by Blazquez-Navarro *et al*.^8^ and 46 (non-reactivated group) without any detectable viral load (Fig. 1).

The overall occurrence of cytomegalovirus reactivation in the cohort ranged 17.58%. None of the patients in the reactivated group had detectable plasma levels of anti-CMV IgM, which confirms reactivation and rules out primary infection. Table 1 describes the baseline characteristics of the groups.

**Table 1:** Characteristics of 56 patients seven days after diagnosis of sepsis with or without cytomegalovirus reactivation.

Variables	All patients N = 56	Non-Reactivated N = 46	Reactivated N = 10	p-value
Age (years)^a^	72 (52–81)	70 (50–83)	74 (69–78)	0.646
Gender^b^				1.000
Male	31 (55)	25 (54)	6 (60)	
Female	25 (45)	21 (46)	4 (40)	
Length of stay in hospital (dias)^a^	17 (13–30)	17 (14–30)	16 (11–34)	0.586
SOFA^a^	6 (4–10)	6 (3–9)	10 (7–11)	0.020
APACHE II^a^	20 (17–25)	20 (17–24)	25 (21–26)	0.124
Glasgow scale^a^	10 (7–15)	10 (7–15)	8 (3–8)	0.037
Sodium (mEq/dL)^a^	139 (136–142)	140 (136–143)	138 (136–141)	0.414
Potassium (mEq/dL)^c^	3.82 ± 0.78	3.77 ± 0.75	4.06 ± 0.91	0.298
Serum bilirubin (mg/dL)^a^	0.4 (0.26–0.7)	0.5 (0.27–0.75)	0.39 (0.24–0.68)	0.656
WBC (mm³)^c^	12833 ± 6574	13163 ± 6940	11345 ± 4562	0.537
Typical lymphocytes^c^	4399 ± 926.9	4366 ± 898	4549 ± 1090	0.575
IL-6 (pg/mL)^a^	10.2 (5.7–19.3)	10.5 (5.8–19.4)	9.9 (5.5–35.2)	0.922
IL-17a (pg/mL)^a^	52.9 (35.5–61.5)	48.7 (32.6–61.6)	55.7 (46.4–62.7)	0.231
Septic shock^b^	35 (63)	26 (57)	9 (90)	0.012
28-days of mortality^b^	17 (30)	11 (24)	6 (60)	0.052
180-days of mortality^b^	32 (57)	24 (52)	8 (80)	0.161

SOFA - Sequential organ failure assessment.

APACHE II - Acute physiology and chronic health evaluation II.

WBC – White blood cells.

IL- interleukin.

^a^Mann Whitney’s U-test, data show as median (25th and 75th percentile).

^b^Fisher’s exact test, absolute number (*n*) (percentage of the group).

^c^Student’s t-test data show as mean ± standart desviation.

The comparison of the groups revealed no statistically significant difference regarding age (p = 0.646), gender (p = 1.000) or length of hospital stay (0.586). In contrast, the SOFA index presented higher values (p = 0.020) while the Glasgow coma scale had lower values (p = 0.037) for the reactivated group.

None of the study patients were in immunosuppressive therapies or clinically considered immunosuppressed. As described in Table 1, the total leukocyte count in both groups remained with an average above 11000/μL and typical lymphocytes above 4100/mm³, with no statistical difference between the groups in any of the variables.

### Increase in nitric oxide metabolites levels and changes in several physiological parameters

**Figure 2:**
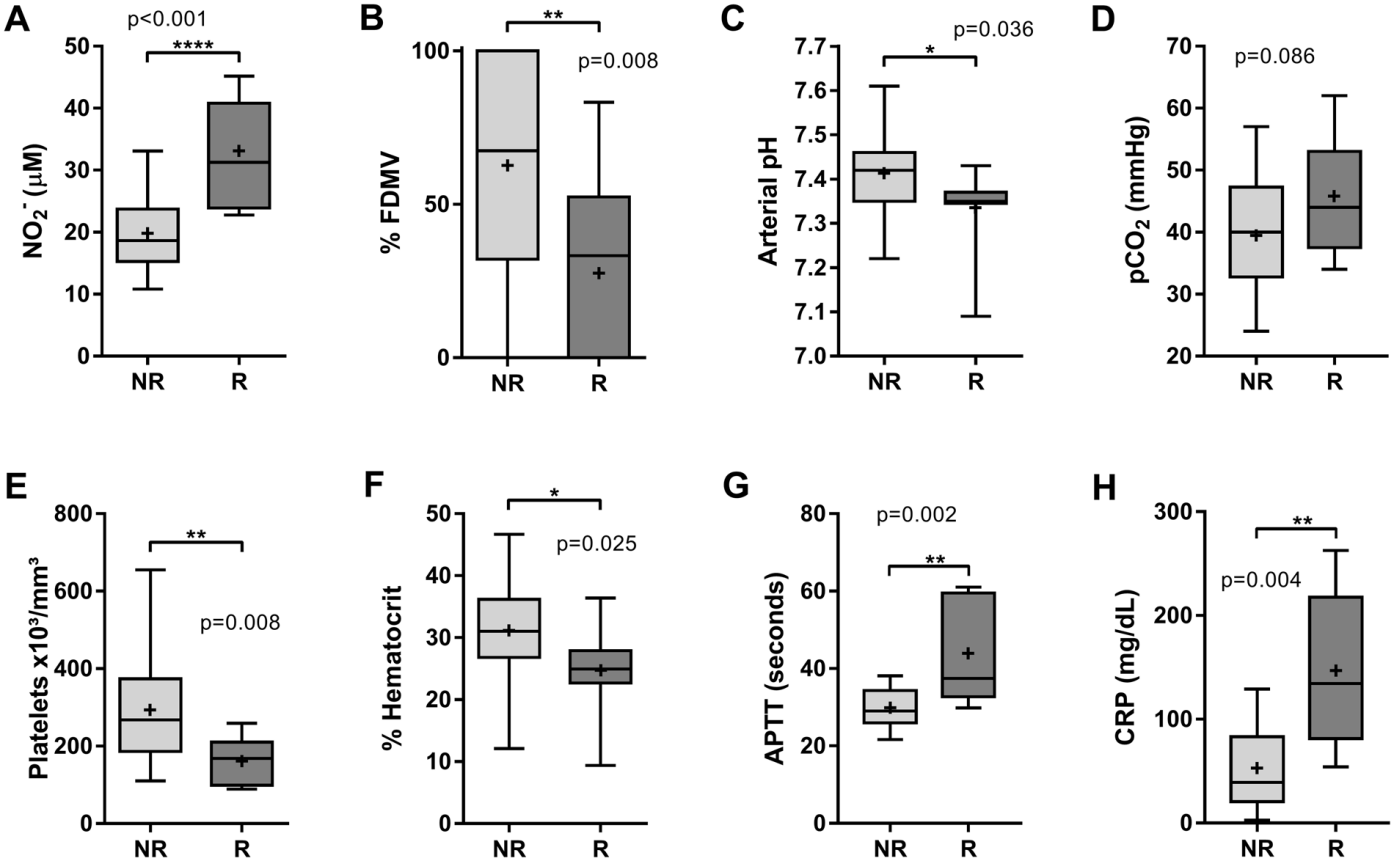
Analysis of clinical and laboratory changes related to CMV reactivation on the seventh day after diagnosis of sepsis. Non-reactivated group (NR). Reactivated group (R). (**A**) levels of plasmatic NO. (**B**) percentage of free days from mechanical ventilation (%FDMV). (**C,D**), arterial pH and partial pressure of arterial carbon dioxide (pCO_2_). (**E**) Counts of total platelets per cubic millimeter x10³. (**F**) Percentage of hematocrit in venous blood. (**G**) activated partial thromboplastin time (APTT), and (**H**) levels of serum C-reactive protein. All data were analyzed through Student’s T test, except %FDMV and % hematocrit which were evaluated by Mann Whitney’s U test. Data show as median at line and mean in +, with 25th and 75th percentile and bars represent the minimum and maximum values.

Plasma levels of NO metabolites proved very high in the reactivated in relation to the non-reactivated group (p < 0.001) (Fig. 2A). In addition, arterial pH (p = 0.036) reduced as well as the percentage of free days from mechanical ventilation (p = 0.008). Partial pressure of arterial carbon dioxide (pCO_2_) levels between the groups did not differ, but a tendency for it to be higher in the reactivated group appeared (p = 0.086) (Fig. 2B–D**)**.

A considerable reduction in total platelet count (p = 0.008) and hematocrit percentage (p = 0.025) also occurred with increased activated partial thromboplastin time (APTT) (p = 0.002), but only for the reactivated group (Fig. 2E–G). Additionally, the inflammatory status in the reactivated group was accentuated by a significant increase in C-reactive protein (CRP) (p = 0.004) (Fig. 2H**)**.

### The increase in IL-10 levels and its association with viral load and renal failure

Subsequently, we evaluated whether CMV reactivation has immunomodulatory activity in sepsis as well as its interaction in the clinical evolution of these patients. The levels of TNF, IFN-γ, IL-2, and IL-4 remained below the detection limits for most of the patients (data not shown). IL-6 and IL-17a did not differ statistically between the groups (p = 0.922 and p = 0.231 respectively).

**Figure 3:**
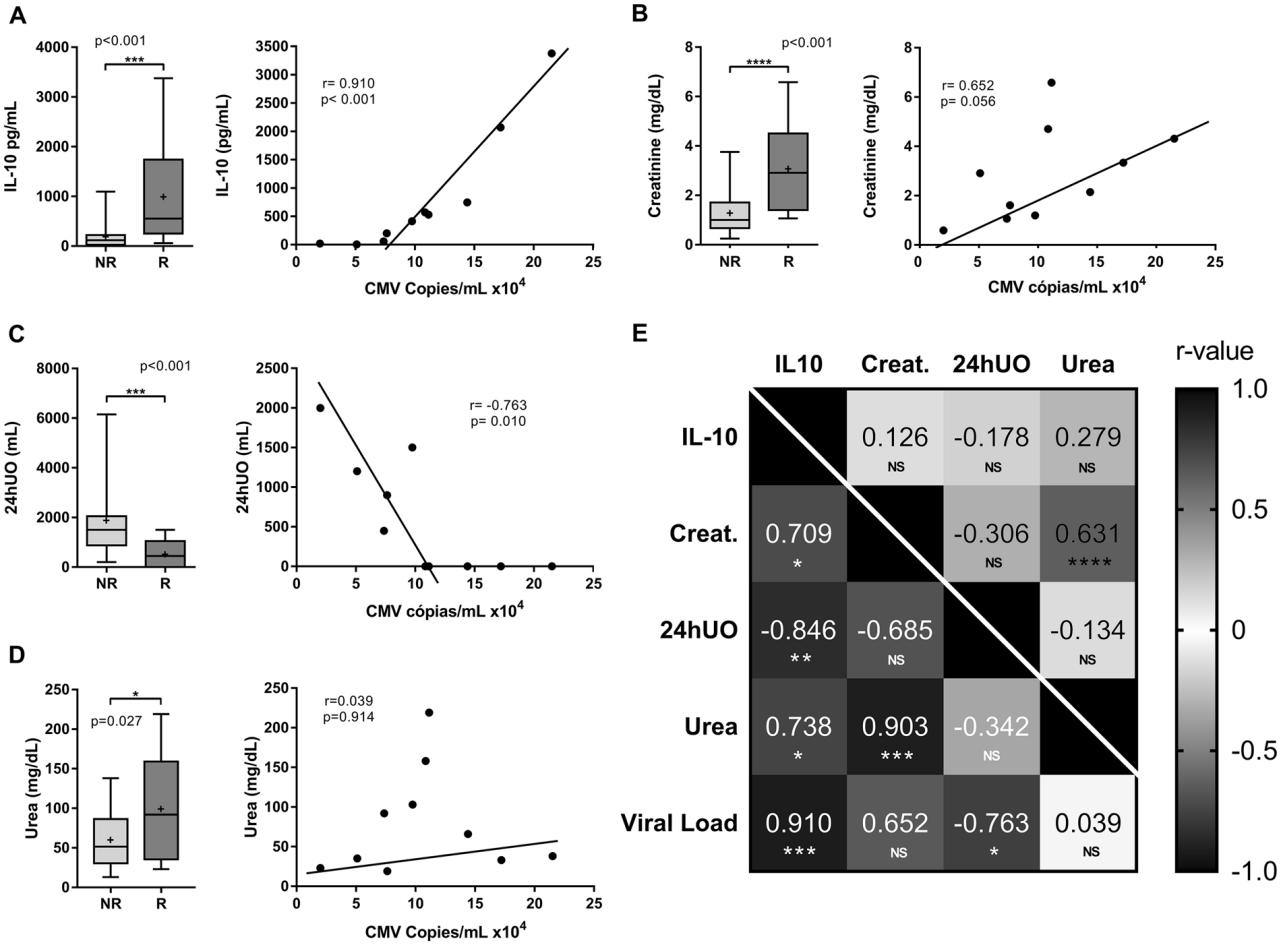
Effect of CMV reactivation on IL-10 levels and renal evaluation parameters. Non-reactivated (NR). Reactivated group (R). Comparison between the groups for plasma level of IL-10 (**A**), serum levels of creatinine (**B**), and urea (**D**), 24-hour urine output (24hUO) (**C**) and its correlation with viral load in the reactivated group. (**E**) Heat map of the r-value through correlation between the variables. Below the cut line are presented the values of the reactivated group and the values of the non-reactivated group, p-values were categorized through NS (p>0.05); ^*^(p ≤ 0.05); ^**^(p ≤ 0.01); ^***^(p≤0.001); ^****^(p≤0.0001). All data were assesed through Student's T test and Pearson’s correlation. Data show as median at line and mean in +, with 25th and 75th percentile and bars represent the minimum and maximum values.

In contrast, we found a significant increase in IL-10 levels for the reactivated group (p < 0.001) with a very strong correlation to CMV viral load (r = 0.910 and p < 0.001) (Fig. 3A). The groups also indicated significant differences regarding the creatinine level and a 24-hour urine output (24hUO) (p < 0.001), directly and inversely correlated to the viral load (r = 0.652/p = 0.056, and r = −0.763/p = 0.010), respectively (Fig. 3B,C). Urea levels also differed between the groups (p = 0.027) with a significant increase for patients with CMV reactivation. A direct correlation appeared between IL-10 and creatinine or urea (r = 0.709/p = 0.026 and r = 0.738/p = 0.045, respectively) in addition to an inverse correlation between IL-10 and 24hUO which occured only in the reactivated group (r = −0.846 and p < 0.001) (Fig. 3E).

### The severity and clinical outcome of the patients

**Figure 4:**
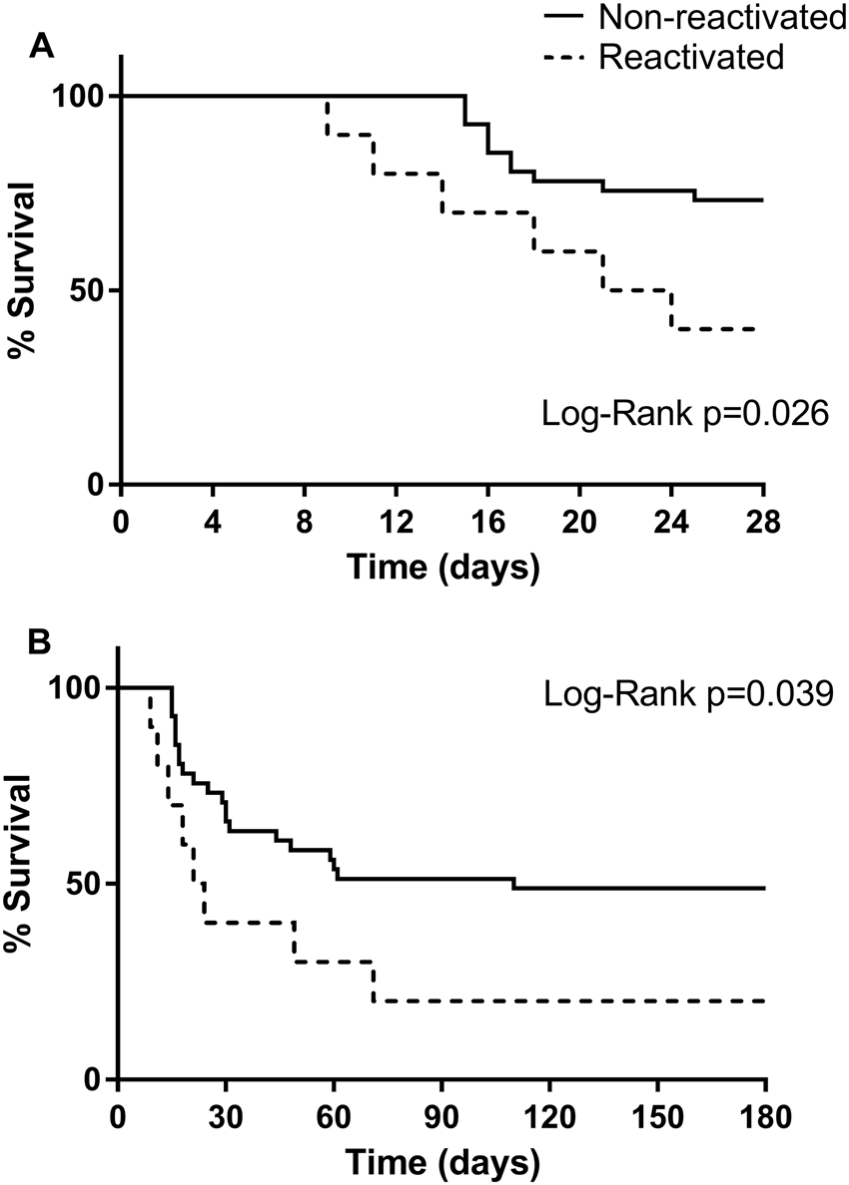
Survival curves among septic patients with or without CMV reactivation. (**A**,**B**) Kaplan-Meier survival analysis (p-value through Log-Rank) comparing septic patients with (dashed line) or without (continuous line) CMV reactivation. (**A**) 28-day mortality. (**B**) 180-day mortality.

Regarding the severity of the clinical condition due to the presence of septic shock, the group with CMV reactivation had a higher incidence (90%) than the non-reactivated group (57%) (RR 2,1; 95%CI1.4–3.1; p = 0.012). A comparison of patients with or without CMV reactivation for morality rates, proved a tendency for statistical difference in 28 (RR 2.5; 95%CI1.2–5.2; p = 0.052), but not in 180 days (RR 1.5; 95%CI1.01-2.3; p = 0.161) post-sepsis diagnosis. However, Kaplan-Meier’s mortality curves differed between groups for both the periods of 28 (log-rank p = 0.026) and 180 days (log-rank p = 0.039) (Fig. 4A,B).

## Discussion

Several studies have clinically demonstrated the effect of CMV reactivation during sepsis, increasing the need for mechanical ventilation and the in-hospital stay of these patients^9^. However, few studies have evaluated the biochemical and immunological variables associated the with viral reactivation which that may influence prognosis and clinical outcome^7^. Our study demonstrated that CMV reactivation changes a variety of laboratory parameters that promote a more intensive decompensation than in patients with sepsis without reactivation, in addition to worsening the general clinical condition evaluated through increased SOFA score and lower consciousness level according to the Glasgow coma scale.

Since we found few studies evaluating cytokines and inflammatory mediators within this sepsis/CMV context. As already described, cytomegalovirus is capable of alters a range of cytokines, so we investigated which could be the most feasible to be measured in our study to investigate how CMV immunomodulation would interfere with sepsis^7,10–12^. So we chose to measure some of the major cytokines of Th1, Th2, and Th17 response patterns, as well as NO.

Our data show an increase in NO and IL-10 levels during CMV reactivation in sepsis, corroborating with several studies pointing out that CMV infection is able to stimulate NO production in several cell types, but such mediator does not act as a microbicidal molecule on this virus. Zhu *et al*.^13^ demonstrated the role of nitric oxide in the escape of the immune system by differentiating hematopoietic progenitor cells into a subtype of long-lived monocytes that produce IL-10, and act as viral reservoir and potent immunosuppressant.

CMV is able to stimulate the expression of excessive NO amounts in the lungs through bronchial epithelial cells, which results in cytotoxicity and injury of adjacent tissue, inducing pneumonitis^14^ and pulmonary fibrosis^15^. Thus, high NO levels observed during reactivation in patients in our study could be implicated in increased respiratory failure and need for mechanical ventilation. Alveolar injury could reduce gas diffusion as well as induce an acid-base imbalance for increase of arterial pCO_2_, resulting in an acidotic state.

The reactivated group had a higher prevalence of septic shock than the control group, suggesting a role of CMV-mediated NO in sepsis severity. CMV has proved to activate NAD(P)H oxidase from arterial smooth muscle cells and deregulate the eNOS expression in endothelial cells, thus contributing to arteriolar vasodilation. In addition, CMV can stimulate venous inflammation and thrombogenic responses^16–18^. Therefore, CMV may be related to the circulatory abnormalities of septic shock; however, further studies are required to elucidate mechanisms possibly involved in this process.

Our study found a considerable decrease in total platelet count and hematocrit percentage, in addition to higher APTT only for the reactivated group. CMV is capable to directly interact and activate platelets through TLR-2 binding, inducing P-selectin expression and the formation of aggregates between platelets and leukocytes via CD169, which stimulates the expression of pro-angiogenic cytokines involved in higher vascular permeability^11,19^.

Hemodynamic disturbances in sepsis may range from a simple subclinical change in blood coagulation to a severe disseminated intravascular coagulation (ICD), characterized by the formation of microthrombi in vessels that contribute significantly to the dysfunction of various organs and consequent consumption of platelets and coagulation factors that may contribute to hemorrhagic manifestations^20,21^. This context suggests that CMV may be able to contribute to the thrombogenic events of sepsis through the direct interaction of platelets and extrinsic pathway activation of blood coagulation due to endothelial damage. Additionally, higher CRP levels also indicate exacerbated inflammatory response to the reactivation process.

IL-10 levels for the reactivated groups were significantly higher and had direct correlation with creatine and urea levels and also a indirect correlation with 24UO. In addition, these variables also showed a correlation with the CMV viral load, with exception of urea.

IL-10 is a key component of the immune system that regulates and suppresses the expression of proinflammatory cytokines during the recovery phases of infections consequently softening the damage caused by inflammatory cytokines^22^. The IL-10 family comprises a set of nine human cytokines and four viral homologs, including one synthesized by the CMV. The cmvIL-10 is secreted by infected cells and binds to cellular IL-10 receptors similarly to the natural ligand, leading to inflammation suppression^23,24^.

In addition, CMV is still capable to induce IL-10 expression by virus-specific CD4^+^T cells upon recognizing MHC-II exhibiting viral antigens. These mechanisms could induce immunosuppression and regulate the inflammatory response, contributing to immune system escape, resistance, and maintenance of CMV infection^23,25^.

Among the diversity of IL-10 functions, this cytokine plays an important role in renal physiology for being related to the development of acute renal diseases and progression to chronic failure^26,27^. The major producers of IL-10 in the kidneys are the mesangial cells, whose abnormal proliferation structurally alters the glomeruli and interstitial tubules and may result in renal failure. IL-10 is a mesangial growth factor with important autocrine activity, promoting cell proliferation. *In vivo* administration of IL-10 in normal rats resulted in considerably higher number of intraglomerular cells and consequent reduction in creatinine clearance. In addition, IL-10 further promotes the deposition of immune complexes that contribute to glomerular injury^24^. Thus, increased IL-10 in patients of the reactivated group may somehow play a role in renal failure in sepsis.

In fact, patients with lower viral load had lower levels of IL-10 and the lower levels of IL-10 in the reactivated group were similar to the median of the non-reactivated group, that is, the levels presented by patients with lowest viral burdens were probably due to sepsis alone and not due to the reactivation process.

Bah *et al*.^28^, demonstrated in murine model the time in days for IL-10 production in sepsis induced by cecal ligation and puncture, and in fact, IL-10 levels were time dependent, with lower levels in the first days after onset of sepsis and Avdic *et al*.^29^ demonstrated the ability of homologous IL-10 produced by cytomegalovirus (cmvIL-10) to immunomodulate and significantly increase the synthesis of human IL-10 (hIL-10) in myeloid cells. The ability of cmvIL-10 to induce the synthesis of hIL-10 was dose dependent, however at lower concentrations this phenomenon was not observed. Thus, it is logical to assume that the higher the viral load, the higher the level of circulating cmvIL-10 and hIL-10 due to this cascade effect and perhaps a low viral load with low cmvIL-10 is not sufficiently capable of triggering this phenomenon or at least in a large-scale, perhaps because of this IL-10 levels in these patients remained similar to the non-reactivated group.

As well as cytomegalovirus, other viruses that have the ability to remain latent may be reactivated in sepsis^30^. Although we did not perform the detection of other pathogens, in our study we demonstrated that the levels of IL-10, creatinine, and 24 h urine output showed a correlation with CMV viral load, which allows us to infer that there is indeed an association and that is with cytomegalovirus and probably not with any other pathogen.

Sepsis is assumed to progress from a primary hyperinflammatory at an early stage to a predominantly immunosuppressive state, with extreme production of pro-inflammatory and anti-inflammatory mediators responsible for a state of immunological dissonance account for refractory shock, multiple organ failure and death^31^.

The overall occurrence of cytomegalovirus reactivation in the cohort ranged 17.58% and the patients had worsening of organ dysfunction, with a 2.1-fold higher risk of developing septic shock, and 2.5-fold higher mortality risk in relation to the group without reactivation within 28 days, in addition to a 33.4% times greater need for mechanical ventilation. In this context, our data suggest an influence of CMV reactivation on the clinical outcome of these patients.

Sepsis may indeed be a trigger factor for cytomegalovirus reactivation that may contribute to worst clinical outcome in these patients, but is not a unique factor, they are two independent phenomena that might have an association, but also have their individualities and factors unrelated to each other that may be involved and contribute to the process. Septic shock may be an event facilitated by cytomegalovirus, since the reactivated group showed a higher prevalence, but is not an exclusive trigger because the non-reactivated group also presented septic shock, but with lower prevalence. It is clear that the low number of individuals in the reactivated group may lead to a sample bias, so we firmly believe that further studies are needed to evaluate this association and to find out if there is a cause and effect relationship.

We demonstrated that CMV is reactivated in sepsis as well as these patients presented a higher risk of developing septic shock and higher mortality rates, especially along the first weeks after reactivation, and our data suggest that IL-10 and NO may be involved in this process. The development kidney failure, respiratory and cardiovascular alterations, as well as changes in the coagulation system and exacerbation of the inflammatory profile, can indicate the possible fields of how CMV reactivation influences the prognosis and clinical outcome of patients with sepsis. Therefore, the development of a better understanding on CMV reactivation in sepsis and possible new strategies to prevent it may contribute significantly to the clinical outcome of these patients.

## Patients and Methods

### Subjects and design

This is a cohort study conducted at the Hospital Universitario de Londrina, Brazil, between May and October 2017. Inclusion criteria: patients of both sexes, aged 18 years, diagnosed with sepsis or septic shock according to sepsis-3 protocol proposed by Singer *et al*.^1^ for a maximum of seven days and serologically positive for anti-CMV IgG. Exclusion criteria: having presented another sepsis within a period of less than three months of the diagnosis used in the study, known immunosuppression, patients suffering from extensive burns and pregnancy. This study was approved by the Committee of Ethics in Research Involving Human Beings of the State University of Londrina (CAAE-61406216.4.0000.5231). All patients who participated in the study or their legal guardians signed a free and informed consent form and were fully informed on all aspects related to the research. All methods performed in the study were conducted following all ethical and legal regulations.

Our initial methodological design was to analyze the variables longitudinally, however, due to the limited sample number, we opted for evaluation only on the seventh day after diagnosis. Limaye *et al*.^32^ demonstrated the time until the appearance of detectable virus load, so this timepoint reflects the beginning of CMV reactivation and allows us to assess early changes associated with it. Knowing that CMV has the capacity to integrate into the host cell and remain in a latency state, we used the cutoff value of viral load > 1000 copies/mL to determine indeed the reactivation, as described by Blazquez-Navarro *et al*.^8^.

Venous blood was collected using two 5 mL tubes containing ethylenediaminetetraacetic acid (EDTA) and the samples were aliquoted for DNA extraction and centrifuged for separation of the plasma which was stored in a freezer at −80 °C until the time of use.

### Determination of plasma levels of anti-CMV IgM and IgG

The detection and quantification of anti-CMV IgM levels were determined through chemiluminescent microparticle immunoassay (Abbott Laboratory, IL, USA) and IgG followed the enzyme immunoassay (ELISA) with the BIOLISA CMV IgG kit (Bioclin, Belo Horizonte, Brazil).

### DNA extraction

The DNA extraction was performed the method described by Green and Sambrook^33^ the aliquots of 200 μL of whole blood were submitted to the classical method of DNA extraction by Phenol-Chloroform. Samples were incubated in lysis buffer (50 mM-TrisHCl-pH 8.0, 50 mM-NaCl, 50 mM-EDTA and 0.5%SDS) with proteinase-K (20 mg/mL) and incubated at 56 °C for 1-hour. Extraction of the organic phase was performed with buffer containing Phenol-Chloroform-Isoamylic Alcohol solution (25:24:1 respectively). The DNA was precipitated in absolute ethanol, eluted in TrisHCl buffer (25 mM-pH 8.0) and stored it in a freezer at −20 °C until use. DNA concentrations were quantified using NanoVue Plus (Biochrom, Holliston, USA) and integrity was assessed using 2% agarose gel electrophoresis.

### Design of oligonucleotide primers and amplification of CMV UL55

Eighty-six DNA sequences from the UL55 region of cytomegalovirus were obtained from the GenBank database available on http://www.ncbi.nlm.nih.gov. The sequences obtained were analyzed using the BioEdit v.7.2.0 software aligned through the ClustalW and a consensus sequence was deduced. From this the primer oligonucleotides were designed and evaluated using the software OligoAnalyzer 3.1. available on https://www.idtdna.com/calc/analyzer. The delineated oligonucleotides (UL55foward 5′-CTGGCATTGCGATTGGTTC-3′ and UL55reverse 5′-CTGTAATCTGAACTGTATGCTGAC-3′) were used in a PCR with a final volume of 20 μl containing 1XPCR buffer, 2.5 mM-MgCl_2_, 1.25 μM each dNTP, 0.65U-Taq DNA polymerase (Invitrogen, Carlsbad, USA), 20 pM of each oligonucleotide and 100 ng of viral genomic DNA. Reactions without the addition of template DNA were performed as negative control. PCR conditions were: 3 minutes at 95 °C, 40-cycles of 94 °C for 15 seconds, 52 °C for 30 seconds and 72 °C for 45 seconds and a final extension of 10 minutes at 72 °C in a Veriti 96-well Thermal Cycler (Applied Biosystems, Foster City, USA). PCR products were evaluated by 2% agarose gel electrophoresis and compared to 100 bp molecular weight marker (Ludwig Biotec, Alvorada, Brazil).

### Cloning and sequencing

Aiming at confirming the specificity of the primers, we cloned the PCR product into plasmid using the vector pGEM®-T Easy (Promega, Madison, USA) according to the manufacturer’s instructions. The cloned plasmids were purified using QIAprep Spin Miniprep kit (Qiagen, Germany) and sequenced using the T7 (foward) and SP6 (reverse) oligonucleotides with ABI BigDye Terminator Kit on an ABI 3500xl Genetic Analyzer Sequencer (Applied Biosystems, Foster City, USA). Electropherograms were analyzed manually on the BioEdit v.7.2.0 software and compared to GenBank sequences using the BLAST algorithm, confirming the primer specificity in the amplification of the 126 base pair fragments.

### Quantification of cytomegalovirus by real-time PCR

For absolute quantification of viral load, a calibration curve was developed based on a 10-fold dilution from 1.6 × 10^6^ and after the real-time PCR (qPCR) assay a linear regression curve was calculated and used to determine the sample viral load, expressed in number of viral copies.

The qPCR was performed on a Rotor-Gene Q 2PlexHRM (Qiagen, USA) thermocycler using QuantiNova SYBR Green PCR kit (Qiagen, USA) and confirmed through Cytomegalovirus (CMV) SYBR Green PCR Kit (Norgen Biotek, Thorold, Canada) with 100 ng/genomic DNA reaction overall, using the forward primer reverse UL55 and UL55 at the concentrations described above. The samples were amplified under the following PCR conditions: an initial step of 2 minutes at 50 °C, 10 minutes at 95 °C and 40 cycles of 30 seconds at 95 °C, 30 seconds at 57 °C and 30 seconds at 72 °C, followed by a test to analyze the dissociation curve (55 °C to 99 °C, with a heating of 0.5 °C/second).

### Plasma cytokine and nitric oxide analysis

Cytometric Bead Array Human Th1/Th2 Cytokine kit (BD Biosciences, San Jose, USA) was used to determine the plasma levels of TNF, IFN-γ, IL-10, IL-6, IL-2, and the IL-17A quantification by Human IL-17A Platinum ELISA (eBioscience, Vienna, Austria). The determination of Nitrite levels as an estimate to produced NO was performed according to Miranda *et al*.^34^.

### Statistical analyses

Statistical analyses were performed using SPSS software (version 20) (IBM Corp, Armonk, USA) and graphs created on Graphpad Prism 7 (GraphPad Software, USA). Data were submitted to the Shapiro-Wilk and Levene tests and those with normal distribution and homogeneity of the variances were evaluated according to the Student’s t-test. Data without normal distribution underwent transformation through natural logarithm; those that nevertheless continued to show no normality or homogeneity were evaluated through the Mann Whitney’s *U*-test. Correlation analyses were performed through the Pearson or Spearman tests, as appropriate. Categorical data were analyzed through Fisher’s exact test and relative risk (RR) with 95% confidence interval (CI). Survival curves followed the Kaplan-Meyer’s method considering 28 and 180 days as cutoff points. Statistical significance was set at p < 0.05 for all analyses.
